# The Clinical and Hematological Markers of Pulmonary Embolism: Multicenter Retrospective Study in Mongolia

**DOI:** 10.3390/hematolrep18050063

**Published:** 2026-08-31

**Authors:** Javzan-Orlom Dorjjugder, Munkh-Erdene Dashzeveg, Zolzaya Batbold, Amgalandari Byambabaatar, Altankhuyag Nergui, Enkh-Amgalan Tserendorj, Yerkyejan Dungyen, Badamsed Tserendorj, Tumur-Ochir Tsedev-Ochir, Batbold Batsaikhan, Solongo Bandi

**Affiliations:** 1 Department of Pulmonology and Allergy, School of Medicine, Mongolian National University of Medical Sciences, Ulaanbaatar 14210, Mongolia; javzaa.shastin@yahoo.com; 2 Department of Pulmonology, Third State Central Hospital, Ulaanbaatar 18061, Mongolia; erdenee0923@gmail.com (M.-E.D.); zolushaa19@gmail.com (Z.B.); amaa.amgalandari@gmail.com (A.B.); altankhuyag.md@gmail.com (A.N.); enhamgalan_tserendorj@yahoo.com (E.-A.T.); ttumurochir@yahoo.com (T.-O.T.-O.); 3 Department of Cardiology, Third State Central Hospital, Ulaanbaatar 18061, Mongolia; erkejan915@gmail.com; 4 Department of Radiology, Third State Central Hospital, Ulaanbaatar 18061, Mongolia; urnaaaa@yahoo.com; 5 Department of Internal Medicine, Institute of Medical Sciences, Ministry of Economy and Development, Ulaanbaatar 13270, Mongolia; batbold.ims@mnums.edu.mn

**Keywords:** systemic inflammation, hematological ratios, NLR, sPESI

## Abstract

**Background/Objectives**: Systemic inflammation-derived indices, including the neutrophil-to-lymphocyte ratio (NLR), platelet-to-lymphocyte ratio (PLR), and systemic immune-inflammation index (SII), have been proposed as convenient and cost-effective predictive markers of mortality and complications in patients with pulmonary embolism (PE). We aimed to evaluate the prognostic performance of hematological inflammatory indices for in-hospital adverse outcomes among Mongolian patients diagnosed with PE. **Methods**: This multicenter retrospective study enrolled patients admitted to three tertiary-level hospitals in Ulaanbaatar, Mongolia, with a confirmed diagnosis of PE between September 2019 and January 2025. Data were collected retrospectively from electronic medical records. We calculated the NLR, PLR, and SII using standard formulas. All patients were stratified according to the sPESI. Moreover, we determined the prognostic performance of hematological indices for in-hospital adverse events. **Results**: We analyzed information on 232 patients with a confirmed diagnosis of PE. According to sPESI scoring, 69.8% (162) were in the high-risk group, with significant differences in comorbidity and age over 60 years (*p* < 0.001). The most common symptoms were dyspnea, chest pain, cough, leg pain, and hemoptysis. The median NLR was 2.48 (1.66–5.08) in the low-risk group and 4.21 (2.04–11.35) in the high-risk group (*p* = 0.001). The median SII level was significantly higher in the high-risk group than in the low-risk group (*p* = 0.037). However, there was no significant difference in PLR level between the two study groups. ROC analysis of hematological indices for predicting in-hospital adverse outcomes was limited, with poor and non-significant discriminatory performance. **Conclusions**: In cases of acute pulmonary embolism, elevated inflammatory indices, NLR and SII, were associated with higher sPESI risk stratification. However, their probability to predict in-hospital adverse events was limited by ROC analysis.

## 1. Introduction

Acute pulmonary embolism (PE) is recognized as the third primary cause of cardiovascular-related deaths globally, following acute myocardial infarction and stroke [1,2]. Geographic differences in PE-related mortality are well documented, suggesting that many unexplained deaths may only be identified as PE during autopsies, indicating systematic underreporting, particularly in low-resource settings [3]. Recent comprehensive registry studies from South Korea, Japan, China, and Taiwan have identified a notable increase in the incidence of PE over the past two decades [4,5]. This trend has been attributed to a variety of factors, including an aging population, lifestyle changes such as the adoption of Western dietary patterns, rising obesity rates, decreased physical activity, a rising prevalence of cancer, and increased availability of CT pulmonary angiography (CTPA) [1]. However, precise prevalence data regarding PE in Mongolia remains insufficient, despite cardiovascular disease being the foremost cause of mortality in the region, and the burden continues to increase [6].

The mortality rate associated with PE can be as high as 30% if timely diagnosis and treatment are not appropriately implemented. The clinical manifestations of PE are often non-specific, typically presenting with symptoms such as dyspnea, pleuritic chest pain, cough, and palpitations, which frequently result in delayed diagnosis across clinical settings worldwide [1]. As reported in the RIETE registry, the 30-day all-cause mortality rate ranged from 4.9% to 6.6%, depending on hemodynamic severity at presentation [7]. Furthermore, low-resource countries’ limited access to D-dimer testing and CTPA results in delayed diagnosis and increased mortality risk, underscoring the need for dedicated PE research in these settings, including Mongolia.

Endothelial injury, dysregulation of the coagulation cascade, and systemic inflammation are fundamental aspects of the pathophysiology of PE [3]. Systemic inflammation-derived indices, including the neutrophil-to-lymphocyte ratio (NLR), platelet-to-lymphocyte ratio (PLR), and systemic immune-inflammation index (SII), are considered straightforward, convenient, and cost-effective predictive markers of mortality and complications in critically ill patients. Many scientists have found that these ratios are potent predictors of mortality and treatment outcome in various conditions, especially in sepsis, cancer, and acute coronary artery syndrome [8,9,10]. However, evidence on the significance of these indices in patients with PE remains limited and inconsistent, and interest among researchers has been increasing. Numerous studies have established that NLR serves as an independent predictor of 30-day mortality in patients diagnosed with PE. Additionally, the SII offers greater discriminative power in this context [11,12,13,14,15].

Risk stratification utilizing the simplified Pulmonary Embolism Severity Index (sPESI) effectively categorizes patients into low-risk (sPESI = 0) and high-risk (sPESI ≥ 1) groups. This classification serves as a predictor of 30-day all-cause mortality and demonstrates performance comparable to that of the original Pulmonary Embolism Severity Index (PESI) score [16,17]. In the current research, we conducted a comparative analysis of clinical, laboratory, and hemodynamic variables between the low-risk and high-risk groups by sPESI score. Furthermore, we aimed to evaluate the prognostic performance of hematological inflammatory indices for in-hospital adverse outcomes among Mongolian patients diagnosed with PE.

## 2. Materials and Methods

### 2.1. Study Design and Setting

This multicenter retrospective study enrolled patients admitted to three tertiary-level hospitals in Ulaanbaatar, Mongolia, all of whom had a confirmed diagnosis of PE (ICD-10 codes I26.0 and I26.9) between September 2019 and January 2025. We included the following tertiary-level hospitals: The First Central Hospital, The Second State Central Hospital, and The Third State Central Hospital.

### 2.2. Study Population

**Figure 1:**
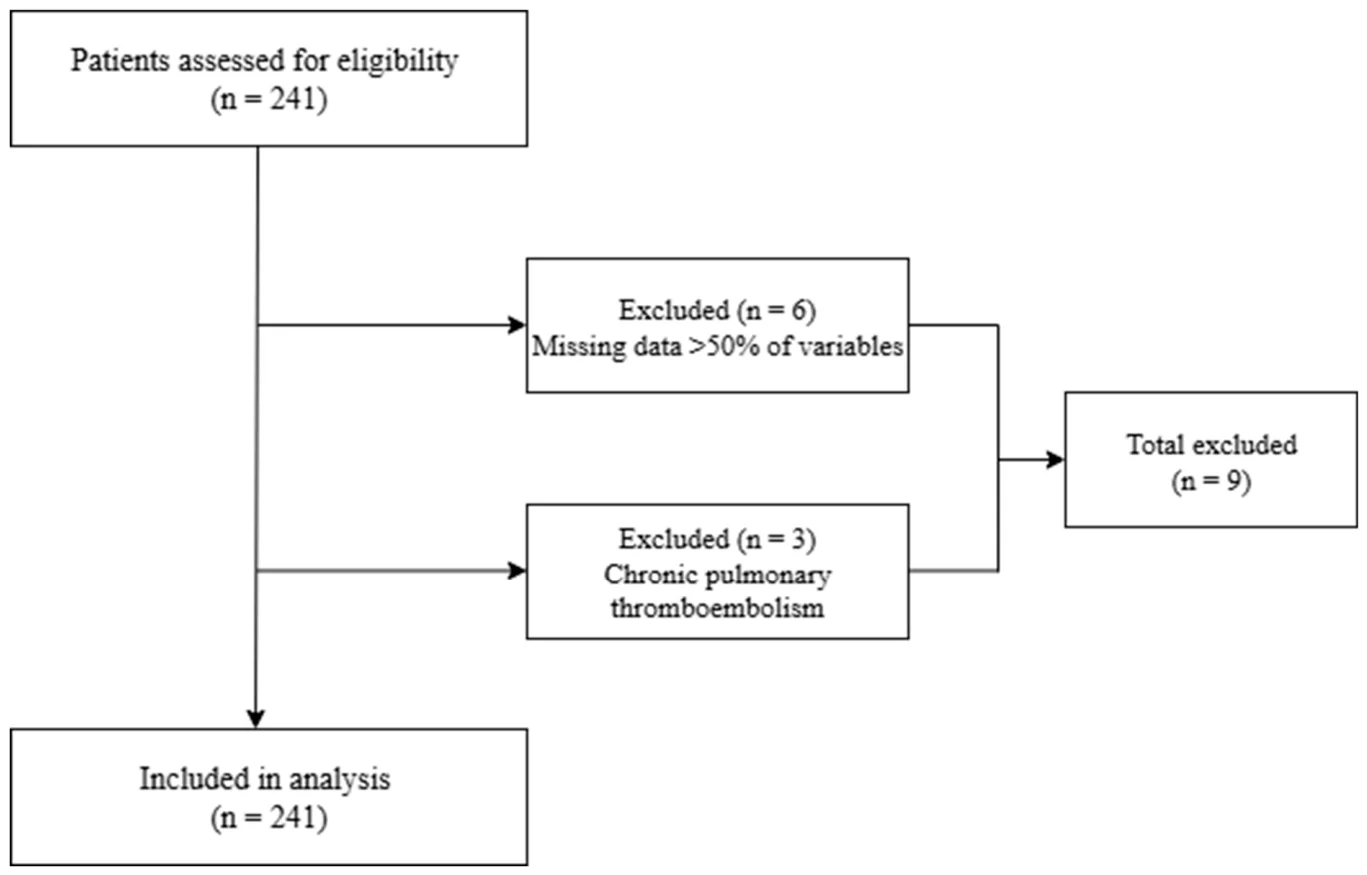
Study participants’ flow chart.

A total of 241 patients with confirmed acute PE were enrolled in the study. We excluded 9 patients based on our eligibility criteria (Figure 1).

We included the following participants: patients with a confirmed diagnosis of PE by CTPA, aged over 18 years, hospitalized at a participating hospital between September 2019 and January 2025, with complete medical records and required laboratory data in the hospital electronic patient history. On the other hand, patients who had missing information for >50% of required clinical or laboratory variables, did not have their diagnosis confirmed by CTPA, and had chronic PE were excluded from this study.

### 2.3. Data Collection

Data were collected retrospectively from hospital archives and electronic medical records. The following information was gathered: demographic characteristics including age, sex, and body mass index (BMI); comorbidities such as arterial hypertension, type 2 diabetes mellitus, obesity, malignancy, chronic obstructive pulmonary disease, heart failure, and previous deep vein thrombosis/pulmonary embolism (DVT/PE); risk factors including immobilization, recent surgery (within the last four weeks), trauma, oral contraceptive use, pregnancy or postpartum status, and history of COVID-19; and presenting symptoms (dyspnea by modified Medical Research Council (mMRC) dyspnea scale, chest pain, hemoptysis, syncope, and lower-limb edema). We also collected hemodynamic parameters at admission, including heart rate (beats/min), systolic and diastolic blood pressure (mmHg), respiratory rate (breaths/min), and peripheral oxygen saturation (SpO_2_, %). From the electronic medical records, we evaluated laboratory parameters, including complete blood count (CBC), white blood cell count, absolute neutrophil, lymphocyte, monocyte, and platelet counts, and serum creatinine and urea.

### 2.4. Inflammatory Index Calculations

The following inflammatory indices were calculated from CBC values obtained at hospital admission:

1. NLR = Absolute neutrophil count ÷ Absolute lymphocyte count.
2. PLR = Platelet count ÷ Absolute lymphocyte count.
3. SII = (Platelet count × Absolute neutrophil count) ÷ Absolute lymphocyte count.

All indices were examined as continuous variables.

### 2.5. Prediction of 30-Day Mortality Using the sPESI

All patients were stratified according to the sPESI, which allocates one point for each of the following criteria: age greater than 80 years, presence of active malignancy, chronic cardiopulmonary disease, a heart rate of 110 beats per minute or more, systolic blood pressure lower than 100 mmHg, and SpO_2_ below 90%. Patients were categorized into two groups based on their 30-day mortality risk using the sPESI score: low-risk (sPESI = 0) and high-risk (sPESI ≥ 1). We compared clinical and laboratory variables between the low-risk and high-risk groups according to the sPESI risk stratification.

### 2.6. In-Hospital Adverse Events

We determined in-hospital adverse events as follows: all-cause death or resuscitated cardiac arrest, and occurrence of hemodynamic instability such as cardiogenic shock or the necessity for positive inotropic or vasoactive drugs. We evaluated the diagnostic accuracy of sPESI and hematological indices to predict in-hospital adverse events.

### 2.7. Statistical Analysis

We performed the analysis using IBM SPSS Statistics version 26.0 (IBM Corp., Armonk, NY, USA). Continuous variables are presented as mean ± standard deviation (SD) or median with interquartile range (IQR), depending on the normality of the distribution assessed by the Shapiro–Wilk test. Categorical variables are represented as frequencies and percentages. In this retrospective study, some variables were not available for all patients due to missing values in medical records. Hence, we performed the analysis using the available data for each variable, and the corresponding denominators are reported in the tables.

To compare groups (low-risk vs. high-risk), we used independent-samples t-tests or Mann–Whitney U tests for continuous variables, and categorical variables were analyzed using the chi-square test or Fisher’s exact test, as appropriate. Receiver Operating Characteristic (ROC) curve analysis was conducted to evaluate the accuracy of the NLR, PLR, SII, and sPESI for predicting in-hospital adverse events. The Area Under the Curve (AUC) with a 95% confidence interval (CI) was calculated for each index. The ROC analysis for sPESI included all 232 patients (in-hospital adverse event group: 21 vs. without in-hospital adverse event group: 211), whereas the ROC analyses of NLR, PLR, and SII included 201 patients with complete data for the corresponding variables and in-hospital adverse-event outcome. A two-tailed *p*-value of less than 0.05 was regarded as statistically significant.

## 3. Results

We analyzed information on 232 patients admitted with a confirmed diagnosis of PE between September 2019 and January 2025. The mean age of participants was 57 ± 17 years; 55.2% (128) were men. According to sPESI scoring, 69.8% (162) were at high risk of death within 30 days, with no significant difference by sex, BMI categories, the presence of DVT, and predisposing factors including long-term immobility and recent surgery. However, patients with comorbidities and those aged over 60 years had significantly higher rates in the high-risk group than in the low-risk group (*p* < 0.001). Compared to the low-risk group, patients with hypertension and other lung diseases had significantly higher proportions in the high-risk group. On the other hand, patients diagnosed with diabetes mellitus 2 and liver disease had significantly higher rates in the low-risk group than in the high-risk group (Table 1).

The most common symptoms were dyspnea (90.9%), chest pain (74.2%), cough (70.5%), leg pain (38.9%), and hemoptysis (20.7%). Patients in the high-risk group more frequently experienced severe dyspnea (*p* < 0.018). However, there was no significant difference in dyspnea severity between the study groups. In the high-risk group, patients experienced cyanosis more frequently, without a significant difference (*p* = 0.134). When we examined vital signs, the patients’ median respiratory rate was higher in the high-risk group than in the low-risk group. Moreover, the median oxygen saturation was 95% (94–98%) in the low-risk group and 91% (88–97%) in the high-risk group, with a significant difference (*p* < 0.001). Half of the study participants were classified into the intermediate-risk group according to both the Wells and Geneva scores. There were no significant differences between groups for the other variables, including heart rate, systolic blood pressure, Wells and Geneva scores, and in-hospital adverse events (Table 2).

We compared the baseline hematological parameters between the two study groups. The median leukocyte and neutrophil counts were significantly higher in the high-risk group than in the low-risk group (*p* = 0.042 and *p* = 0.02, respectively). Moreover, the median hemoglobin and hematocrit levels were significantly higher in the high-risk group at 14 (12–16) g/dL and 42.3% (35.8–47.8%), respectively (*p* = 0.044 and *p* = 0.012). On the contrary, the median platelet count was 278 (197–470) in the high-risk group and 212 (166–300) in the low-risk group, with a significant difference (*p* < 0.001). We found a significantly lower lymphocyte count in the high-risk group at 1.47 (0.85–2.36), with a *p*-value of <0.001. When renal function was assessed, the median levels of creatinine and BUN were significantly higher in the high-risk group than in the low-risk group (*p* < 0.001). We calculated NLR, PLR, and SII according to the standard formulas. The median NLR was 2.48 (1.66–5.08) in the low-risk group and 4.21 (2.04–11.35) in the high-risk group, with a significant difference (*p* = 0.001). The median SII level was significantly higher in the high-risk group than in the low-risk group (*p* = 0.037). However, there was no significant difference in PLR level in the two study groups (Table 3).

A total of 21 cases were recorded as the in-hospital adverse event group, including cases of all-cause death or resuscitated cardiac arrest and occurrence of hemodynamic instability such as cardiogenic shock or the necessity for positive inotropic or vasoactive drugs. ROC analyses of NLR, PLR, and SII included 201 patients with complete data, comprising 19 with adverse events and 182 without. In this analysis, we excluded 31 patients because of missing data. Conversely, we performed the ROC curve analysis of sPESI using the entire study population, including 21 patients with in-hospital adverse events and 211 patients without adverse events. We estimated the diagnostic accuracies of NLR, PLR, SII, and sPESI and determined optimal cut-off values using Youden’s index. The sensitivity and specificity of NLR in predicting in-hospital adverse events were 42.1% and 80.2%, respectively, at a cut-off of 7.52 (AUC = 0.554, 95% CI 0.402–0.707). The PLR and SII cut-off values for predicting in-hospital adverse events were determined to be 166.15 and 1795.67, respectively, with sensitivities of 52.6% and 47.4% and specificities of 58.3% and 77.8%, respectively. The ROC curve for sPESI as a predictor of in-hospital adverse events showed an AUC of 0.680 (95% CI, 0.553–0.807) at a cut-off > 1.5, with a sensitivity of 57.1% and a specificity of 72.0% (Table 4).

**Figure 2:**
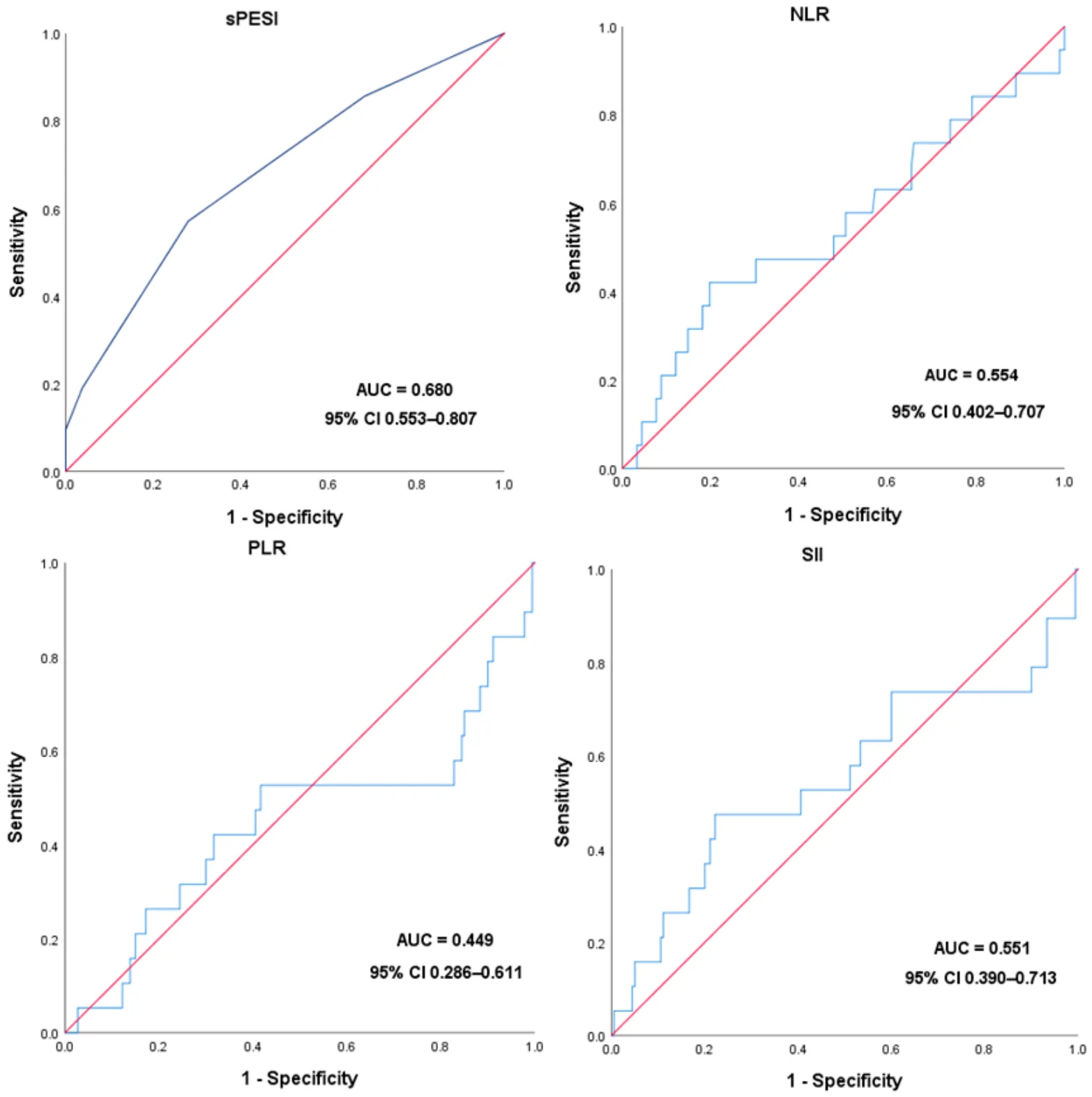
ROC curves for sPESI and other hematological markers as a predictor of in-hospital adverse events.

In Figure 2, we illustrate the diagnostic accuracy of sPESI and other hematological markers. sPESI showed a slightly higher AUC than the other hematological indices evaluated (Figure 2).

## 4. Discussion

We found that elevated inflammatory indices, NLR and SII, were associated with higher sPESI risk stratification. However, their ability to predict in-hospital adverse events was limited by ROC analysis. Across numerous studies, dyspnea, chest pain, cough, and hemoptysis are the predominant symptoms of PE. As stated in reviews and cohort studies, dyspnea is reported in roughly 73–85% of cases, chest pain in 39–64%, cough in 28–43%, and hemoptysis in 5–23% [18,19,20,21]. The lower oxygen saturation levels and elevated respiratory rates observed in the high-risk sPESI group demonstrate a well-established correlation between hypoxemia, tachypnea, and the significant hemodynamic consequences associated with PE [20,21,22]. Our findings of frequent dyspnea (90.9%), chest pain (74.2%), cough (70.5%), and hemoptysis (20.7%) are consistent with this pattern.

We found that age was significantly higher in high-risk patients, likely due to associated comorbidities that influence the prognosis of acute PE. Deep vein thrombosis was the main cause of acute PE in our study. However, some studies have found that active malignancy is a main risk factor for DVT [23,24]; we did not find this correlation. This is because the included hospitals did not regularly provide healthcare services for patients with cancer or undergoing cancer therapy.

Systematic inflammation and coagulation disturbances play a crucial role in increasing the risk of venous thromboembolism, including pulmonary embolism [25]. The coagulation imbalance is characterized by increased procoagulant activity, fibrinogen, and D-dimers and reduced endogenous anticoagulants such as antithrombin III and protein C [26,27]. Mitroi DM et al. found that patients with tuberculosis and reduced levels of natural anticoagulants such as protein S and protein C were associated with a higher incidence of pulmonary embolism [28]. In our study, however, we did not evaluate natural anticoagulants. Nevertheless, patients in the high-risk sPESI group had significantly lower platelet (PLT) counts compared with those in the low-risk group.

Neutrophilia and lymphocytopenia are immune responses that occur in reaction to systemic inflammation, injury, and stress. These conditions are influenced by a variety of factors that warrant careful consideration [29]. The prompt response of neutrophil and lymphocyte counts within the first six hours following acute physiological stress positions them as early indicators, particularly when compared to other laboratory parameters such as the white blood cell count and C-reactive protein levels [30]. However, the interval between symptom onset and admission could not be evaluated. In our study, the median NLR at admission in PE patients was 3.70 (1.94–8.29), higher than that of healthy individuals, with a normal range of 1–2 reported in the review study [31]. In the original study, NLR values below five are classified as normal. However, it is important to note that distinct cut-off values have been identified for various diseases, including malignancy, sepsis, and cardiovascular conditions. Currently, there is no consensus on a standardized pathological value across these differing contexts.

In circumstances that represent significant triggers for systemic inflammatory responses, such as sepsis, malignancy, rheumatologic disorders, and trauma, there is not only an increase in neutrophilia and lymphopenia, but there is also an induction of platelet proliferation. This phenomenon is primarily driven by pro-inflammatory cytokines, particularly interleukin-6 (IL-6) and interleukin-1 (IL-1) [32,33]. In our study, we did not find a significant correlation between PLR levels and the two study groups, nor did we find potent diagnostic accuracy for in-hospital adverse events. This is consistent with the meta-analysis, which included 15 studies that found that NLR was a significant predictor of mortality in patients with PE, but PLR was not. The included studies in this meta-analysis reported NLR cut-offs ranging from 3.25 to 8.4 and PLR cut-offs from 170 to 325 [34]. In our study, we used a cut-off value of 7.52 to predict PE-related adverse events, achieving an AUC of 0.554. These cut-off values are derived from our study and should not be interpreted as clinically established thresholds. As stated in recent studies, more reliable performance of the NLR compared with the PLR and SII has been observed in various conditions, including ischemic stroke, pneumonia following intracerebral hemorrhage, and infective endocarditis. NLR appears to be the most robust of these indices; however, it is essential to consider it within the appropriate clinical context for accurate interpretation [35,36,37]. Nevertheless, our findings did not demonstrate a similar trend, as NLR did not show superior predictive performance compared with PLR or SII in our study population.

Numerous validation studies conducted across diverse populations have demonstrated that the sPESI effectively identifies low-risk patients, with a 30-day mortality rate of less than 1%. Furthermore, it performs as well as, or even surpasses, other clinical prognostic tools in this regard [38,39,40]. We find that an AUC of 0.68 for sPESI, with moderate sensitivity and specificity for adverse in-hospital events, is consistent with external data, which typically report AUCs in the 0.70 range [40,41]. A comprehensive review of the critical care literature indicates that these biomarkers have prognostic significance across various inflammatory conditions. However, it is important to note that their predictive capabilities are limited when considered in isolation. They tend to yield more accurate insights when utilized in conjunction with clinical scoring systems or other biomarkers [30].

Several important limitations must be acknowledged in this study. Firstly, the sample size, particularly within the adverse-event subgroup (n = 21), limits the statistical power of the ROC analyses and may lead to underestimation or overestimation of the prognostic value of the NLR, PLR, and SII. Secondly, the assessment of NLR, PLR, and SII was conducted solely at baseline. Prior research indicates that dynamic changes over time could provide valuable prognostic information that was not evaluated in this study. Therefore, it is essential to conduct large-scale prospective cohort studies to further clarify this association.

## 5. Conclusions

In the context of acute pulmonary embolism, elevated levels of NLR and SII are associated with the higher sPESI risk category. ROC analysis of hematological indices for predicting in-hospital adverse outcomes was limited, with poor and non-significant discriminatory performance. Larger prospective studies are needed to establish robust evidence regarding the prognostic value of these hematologic indices for predicting early complications and to determine their role in improving the early detection and management of PE, especially in low- and middle-resource countries.

## Figures and Tables

**Table 1:** Study participants’ demographic characteristics by sPESI classification.

Variables *	sPESI Classifications		Total	*p* Value
	Low Risk	High Risk		
	Count (Percent)			
Sex (n = 232)				
Male	38 (54.3%)	90 (55.6%)	128 (55.2%)	0.858
Female	32 (45.7%)	72 (44.4%)	104 (44.8%)	
BMI (n = 222)				
Normal	24 (35.8%)	56 (36.1%)	80 (36.0%)	0.615
Overweight	26 (38.8%)	51 (32.9%)	77 (34.7%)	
Obesity	17 (25.4%)	48 (31.0%)	65 (29.3%)	
Comorbidity (yes) (n = 225)	30 (45.5%)	144 (90.6%)	174 (77.3%)	<0.001
Comorbidities (n = 177)				
Diabetes Mellitus-2	6 (18.8%)	7 (4.8%)	13 (7.3%)	0.02
Hypertension	2 (6.3%)	75 (51.7%)	77 (43.5%)	<0.001
CKD	2 (6.3%)	1 (0.7%)	3 (1.7%)	0.26
Heart Disease	0 (0.0%)	21 (14.5%)	21 (11.9%)	0.11
Other lung diseases	1 (3.1%)	27 (18.6%)	28 (15.8%)	0.04
Cancer	0 (0.0%)	3 (2.1%)	3 (1.7%)	0.98
Liver disease	3 (9.4%)	0 (0.0%)	3 (1.7%)	0.02
Digestive diseases	2 (6.3%)	3 (2.1%)	5 (2.8%)	0.6
Rheumatologic diseases	2 (6.3%)	3 (2.1%)	5 (2.8%)	0.6
Other	14 (43.8%)	5 (3.4%)	19 (10.7%)	<0.001
Constant drug use (yes) (n = 224)	19 (27.9%)	116 (74.4%)	135 (60.3%)	<0.001
History of surgery (yes) (n = 230)	16 (23.2%)	24 (14.9%)	40 (17.4%)	0.129
COVID-19 infection (yes) (n = 191)	23 (40.4%)	59 (44.0%)	82 (42.9%)	0.638
Cancer therapy (yes) (n = 178)	0 (0.0%)	5 (3.9%)	5 (2.8%)	0.324
DVT (yes) (n = 232)	40 (57.1%)	90 (55.6%)	130 (56.0%)	0.823
Immobility (yes) (n = 181)	11 (20.4%)	16 (12.6%)	27 (14.9%)	0.179
Age	48 ± 14	60 ± 15	57 ± 16	<0.001

* Values are presented as n (%). Denominators vary across variables because of missing data; percentages are calculated based on available observations for each variable.

**Table 2:** Study participants’ clinical characteristics and physical examination by sPESI classification.

Variables *	sPESI Classifications		Total	*p* Value
	Low Risk	High Risk		
	Count (Percent)			
Dyspnea (yes) (n = 231)	58 (84.1%)	152 (93.8%)	210 (90.9%)	0.018
mMRC (n = 183)				
mMRC0	0 (0.0%)	6 (4.4%)	6 (3.3%)	0.162
mMRC1	13 (27.1%)	24 (17.8%)	37 (20.2%)	
mMRC2	25 (52.1%)	57 (42.2%)	82 (44.8%)	
mMRC3	8 (16.7%)	39 (28.9%)	47 (25.7%)	
mMRC4	2 (4.2%)	9 (6.7%)	11 (6.0%)	
Cough (yes) (n = 227)	46 (67.6%)	114 (71.7%)	160 (70.5%)	0.54
Hemoptysis (yes) (n = 227)	18 (26.5%)	29 (18.2%)	47 (20.7%)	0.161
Chest pain (yes) (n = 229)	55 (79.7%)	115 (71.9%)	170 (74.2%)	0.214
Cyanosis (yes) (n = 226)	3 (4.4%)	18 (11.3%)	21 (9.3%)	0.134
Leg pain (yes) (n = 229)	27 (39.1%)	62 (38.8%)	89 (38.9%)	0.957
Respiratory rate (per min) (n = 220)	19 (18–23)	20 (18–23)	20 (18–23)	0.001
Heart rate (per min) (n = 232)	81 (74–94)	85 (75–102)	84 (74–98)	0.076
Oxygen saturation (n = 231)	95 (94–98)	91 (88–97)	94 (88–97)	<0.001
Systolic blood pressure (n = 232)	120 (110–130)	120 (100–140)	120 (110–140)	0.757
Wells score (n = 214)				
Low risk	20 (31.7%)	38 (25.2%)	58 (27.1%)	0.614
Intermediate risk	33 (52.4%)	87 (57.6%)	120 (56.1%)	
High risk	10 (15.9%)	26 (17.2%)	36 (16.8%)	
Geneva score (n = 222)				
Low risk	19 (29.2%)	38 (24.2%)	57 (25.7%)	0.511
Intermediate risk	33 (50.8%)	93 (59.2%)	126 (56.8%)	
High risk	13 (20.0%)	26 (16.6%)	39 (17.6%)	
Adverse events (yes) (n = 232)	3 (4.3%)	18 (11.1%)	21 (9.1%)	0.134

* Values are presented as n (%). Denominators vary across variables because of missing data; percentages are calculated based on available observations for each variable.

**Table 3:** Baseline hematologic parameters by sPESI classification.

Variables	sPESI Classifications		Total	*p* Value
	Low Risk	High Risk		
	Median (IQR)			
Leukocytes (×10^3^/μL) (n = 226)	8.24 (5.85–13.21)	8.95 (6.51–14.55)	8.70 (6.40–13.72)	0.042
Hemoglobin (g/dL) (n = 225)	13.3 (11.2–15.6)	14 (12–16.2)	13.9 (11.9–15.8)	0.044
Hematocrit (%) (n = 222)	39.4 (34–46)	42.3 (35.8–47.8)	41.7 (35.8–47.1)	0.012
Platelets (×10^3^/μL) (n = 225)	278 (197–470)	212 (166–300)	235 (178–325)	<0.001
MPV (fl) (n = 187)	9.35 (8.7–10.4)	9.7 (8.9–10.4)	9.6 (8.9–10.4)	0.143
Neutrophils (×10^3^/μL) (n = 205)	5.535 (2.99–10.24)	6.44 (3.99–12.37)	6.25 (3.73–9.95)	0.02
Lymphocytes (×10^3^/μL) (n = 201)	1.99 (1.3–3.02)	1.47 (0.85–2.36)	1.61 (1.00–2.61)	<0.001
NLR (n = 201)	2.48 (1.66–5.08)	4.21 (2.04–11.35)	3.70 (1.94–8.29)	<0.001
PLR (n = 199)	146.98 (78.12–290.53)	152.27 (85.75–297.86)	147.83 (89.79–277.44)	0.444
Creatinine (µmol/L) (n = 198)	67.45 (51.27–105)	81.75 (60–122.2)	75.65 (57.47–112.27)	0.001
BUN (mmol/L) (n = 175)	4.71 (3.3–9.6)	6.805 (4.4–13.41)	6.42 (4.00–12.30)	0.001
SII (n = 199)	765.7 (442.5–1380.3)	980.5 (431.4–2626.2)	920.3 (442.5–2289.4)	0.037

**Table 4:** Optimal cut-off levels of hematologic indices to predict in-hospital adverse events.

Variables	Youden Cut-Off Value	AUC	Sensitivity	Specificity	CI
NLR	7.52	0.554	42.1%	80.2%	0.402–0.707
PLR	166.15	0.449	52.6%	58.3%	0.286–0.611
SII	1795.67	0.551	47.4%	77.8%	0.390–0.713
sPESI	1.5	0.680	57.1%	72.0%	0.553–0.807

## Data Availability

The datasets presented in this article are not readily available due to technical and time constraints in the database.

## Abbreviations

The following abbreviations are used in this manuscript:

PE	Pulmonary embolism
CTPA	Computed tomography pulmonary angiography
NLR	Neutrophil-to-lymphocyte ratio
PLR	Platelet-to-lymphocyte ratio
SII	Systemic inflammation index
sPESI	Simplified pulmonary embolism severity index
PESI	Pulmonary embolism severity index
ROC	Receiver operating curve
AUC	Area under curve
BUN	Blood urea nitrogen
